# Biological sex modulates lung injury severity in adolescent mice exposed to short‐term aerosolized vitamin E acetate

**DOI:** 10.14814/phy2.70691

**Published:** 2025-12-11

**Authors:** Michelle J. Lim, Xiaohan Li, Jihau Yu, Heesun Kim, Delaney Stevenson, Negin Esfandiari, Lillian N. Tran, Tran B. Nguyen, Jocelyn A. Livezey, Christoph F. A. Vogel, Eliot R. Spindel, Timothy E. Albertson, Michael A. Matthay, Kent E. Pinkerton

**Affiliations:** ^1^ Department of Pediatrics, Division of Critical Care UC Davis Children's Hospital, UC Davis School of Medicine Sacramento California USA; ^2^ Center for Health and the Environment University of California Davis California USA; ^3^ Department of Environmental Toxicology University of California Davis California USA; ^4^ Department of Pediatrics UC Davis School of Medicine Sacramento California USA; ^5^ Division of Neuroscience, Oregon National Primate Research Center Oregon Health & Science University Beaverton Oregon USA; ^6^ Department of Internal Medicine, Division of Pulmonary and Critical Care Medicine UC Davis Medical Center, UC Davis School of Medicine Sacramento California USA; ^7^ Department of Medicine and Anesthesia, Cardiovascular Research Institute University of California San Francisco California USA

**Keywords:** adolescence, E‐cigarette or vaping product use‐associated lung injury, EVALI, inflammation, vitamin E acetate

## Abstract

The E‐cigarette or Vaping product‐Associated Lung Injury (EVALI) causes severe acute respiratory failure and, in some cases, death. Most cases are linked to tetrahydrocannabinol‐containing e‐cigarette products adulterated with vitamin E acetate. Despite regulation and awareness efforts, VEA persists in biological samples from EVALI patients and remains a public health concern, particularly among adolescent males. The mechanisms driving VEA‐induced lung injury, and how they may be differentiated by sex, remain poorly understood. To address this, age‐ and size‐matched adolescent male and female mice were exposed to aerosolized VEA for 3 or 10 days. By Day 10, VEA exposure caused histopathologic lung injury and systemic inflammation, with alveolar barrier dysfunction evident on Day 3. Male mice developed more severe injury and immune dysregulation, with elevated lung interleukin‐1β, interleukin‐6, and keratinocyte chemoattractant and reduced expression of club cell secretory protein along the airway epithelium. Female mice showed higher serum levels of soluble receptor for advanced glycation end products, a biomarker of alveolar injury and inflammation that also functions as an immune modulator. This is the first study to identify sex‐specific differences in pulmonary responses to VEA exposure. These findings offer insight into EVALI immunopathogenesis and may explain population‐level sex disparities in disease severity.

## 1 INTRODUCTION

First recognized in 2019, E‐cigarette or Vaping product use–Associated Lung Injury (EVALI) is a serious and potentially fatal pulmonary illness linked to the use of e‐cigarettes or vaping products (Cao et al., 2020; Henry et al., 2020; Layden et al., 2020; Maddock et al., 2019; Schier et al., 2019). The condition remains a clinical challenge due to the absence of specific diagnostic criteria, limited understanding of its underlying pathophysiology, and insufficient data on its true current incidence and prevalence (Jatlaoui et al., 2019; Schier et al., 2019).

During the 2019 EVALI outbreak, the Centers for Disease Control and Prevention (CDC) reported 2807 hospitalizations and 68 deaths across the United States over a six‐month period (Rebuli et al., 2023). The epidemic showed a disproportionate number of males affected by severe disease and higher susceptibility among younger individuals, especially adolescent youth but the actual sex‐related incidence remains unknown (Bao et al., 2020; Ellington et al., 2020; Miech et al., 2019; Werner et al., 2020). The majority of individuals diagnosed with EVALI at the time had used tetrahydrocannabinol (THC)‐containing products adulterated with vitamin E acetate (Barrot et al., 2020), a synthetic compound of vitamin E that resembles THC in appearance and is commonly used as a cutting agent to prolong product use (Duffy et al., 2020; Taylor et al., 2019). VEA was detected in the bronchoalveolar lavage (BAL) fluid of 94.1% of hospitalized patients diagnosed with EVALI across 16 U.S. states during the 2019 outbreak (Blount et al., 2020). Given this strong epidemiologic association, VEA was widely assumed to be the primary causative agent of EVALI (Arons et al., 2021).

VEA is distinguished by its high viscosity and low thermal stability compared to common e‐cigarette solvents. Under higher temperatures, VEA can degrade into ketene, a highly reactive and toxic gas capable of inducing protein acetylation and promoting lung cellular cytotoxicity (Strongin, 2020). However, the precise pathophysiologic role of VEA in inducing acute lung injury remains unknown (Rebuli et al., 2023). Further, it is unclear whether the observed gender disparities associated with VEA susceptibility and disease severity are rooted in biological sex differences and sex‐based variation in immune response. Since the initial outbreak, public and scientific attention to EVALI has waned, hindering deeper investigation into its disease mechanisms and broader impact on population health. Nevertheless, EVALI remains a pressing public health concern as cases continue to be reported and the use of vaping products particularly among youth remains widespread (Amjad et al., 2025). In addition, significant gaps persist in FDA oversight of both cannabinoid and e‐cigarette products, and the long‐term health sequelae in survivors remain unknown (Callahan et al., 2024; Cao et al., 2020; McAlinden et al., 2020; Rebuli et al., 2023).

In this study, we sought to further characterize the early pathobiological effects and onset of lung injury following short‐term, intermittent aerosolized VEA exposure over 3‐ and 10‐day periods in a novel murine model of young adulthood that is naïve to prior inhaled toxicants and free of preexisting lung disease. We also aimed to elucidate the cellular and molecular mechanisms underlying EVALI pathogenesis by profiling mRNA expression of key pro‐inflammatory cytokines and chemokines implicated in acute lung injury, along with quantification of plasma soluble receptor for advanced glycation end products (sRAGE), a marker of alveolar epithelial injury and inflammation, and club cell secretory protein (CCSP) expression, a surrogate marker of airway epithelial injury. Finally, we sought to perform a sex‐stratified analysis to identify potential sex–specific differences with EVALI pathogenesis.

## 2 MATERIALS AND METHODS

### 2.1 Animals and exposure conditions

All experimental procedures were performed under protocols approved by the UC Davis Institutional Animal Care and Use Committee (IACUC). C57BL/6 male and female mice (Inotiv, West Lafayette, IN) were acclimated for 2 weeks prior to the exposure, maintained on a 12‐h light/dark cycle, and housed three per cage using sterile laboratory bedding and access to food (Cat# 15001, Newco, Rancho Cucamonga, CA) and water ad libitum. At 10 weeks of age, mice were randomly assigned to exposure to filtered air (control) or VEA aerosol in a whole‐body exposure chamber (Teague Enterprises, Woodland, CA). A total of four exposure groups were performed including male 3‐day, female 3‐day, male 10‐day, and female 10‐day with each group having *N* = 6 for the VEA and corresponding control groups. The mice were exposed to VEA aerosol for 3 h/day. The body weight of each animal was recorded immediately prior to and after each exposure duration.

### 2.2 Inhalation chamber setup, aerosol generation, and analysis

The aerosol was generated using undiluted VEA (DL‐α‐Tocopherol acetate; >96% HPLC, Sigma‐Aldrich, St. Louis, MO) and a third‐generation Evolv DNA 75 modular device (Evolv LLC, Hudson, OH) with a refillable tank and single‐mesh coil (SS316L, FreeMax, Inc., Shenzhen, China). The device was operated at a power of 55 W and maintained a puffing regime of 3 s/puff and 2 puffs/min by an external controller (Teague Enterprises, Woodland, CA). The generated aerosol was drawn into and exited the exposure chamber by a vacuum source at a flow rate of 5 L/minute.

The aerosol concentration inside the chamber was measured three times at 45‐min intervals per 3‐h exposure. The chamber aerosol was collected onto 25 mm Pallflex® Emfab™ filter papers (Pall Corporation, Port Washington, NY) using a filter housing unit (Teague Enterprises, Woodland, CA). Each sample was collected for 10 min at a flow rate of 1 L/min. Filter papers were weighed pre‐ and post‐ collection, respectively. The aerosol concentration was calculated as follows:

$$\begin{array}{c} \text{Aerosol concentration}\;\left(\mathrm{mg}/m^{3}\right)\\ \;=\frac{\left(\text{post}-\text{collection mass}\right)-\left(\mathrm{pre}-\text{collection mass}\right)}{\text{Total volume of collection}\;}. \end{array}$$

The daily chamber concentration of VEA was created to represent overall puff conditions generated by a third‐generation e‐cigarette device under normal operation. The goal was to establish a consistent overall concentration of VEA aerosol that could potentially mimic human exposure conditions for both male and female mice.

### 2.3 Physiological measurements of blood oxygen saturation, respiratory rate, and heart rate

A MouseOx® Plus, a mouse pulse oximeter, (STARR Life Sciences, Oakmont, PA) was used to measure breath rate, heart rate, and oxygen saturation levels. Pulse oximeter measurements were taken following 4 and 9 days of the 10‐day studies in both male and female mice (Bi et al., 2021). To facilitate these measurements, mice were shaven around the neck, followed by acclimation to the pulse oximeter collar, placed dorsally around the neck, for 3 h prior to pulse oximeter measurements. The mice were anesthetized to place the neck collar sensor and allowed to recover for 5 min prior to the start of measurements. Vital measurements were collected and monitored for 10 min. Each mouse was housed in a small animal enclosure during the measurements.

### 2.4 Bronchoalveolar lavage fluid (BALF)

The mice were weighed and euthanized at the end of each exposure. In this study, the term “necropsy” refers to postmortem collection and analysis of biological samples from these animals. Prior to the necropsy, the animals were euthanized by 0.3 mL of sodium pentobarbital (55.7 mg/mL) via intraperitoneal injection. The trachea was cannulated to allow left lung fixation and BALF collection. The BALF was lavaged from the right lung while the main bronchus of the left lung was clamped using two aliquots of 0.8 mL Dulbecco's phosphate‐buffered saline (PBS; Sigma‐Aldrich, St. Louis, MO) with each being instilled three times prior to final collection. The supernatant was separated for total protein quantification and chemical analysis. BALF cell viability and concentration were determined using trypan blue solution (Sigma‐Aldrich, St. Louis, MO) and a hemocytometer (Bright‐Line, Hausser Scientific, Horsham, PA) under a light microscope.

The cytospin slides were prepared by centrifuging 100 μL of resuspended BAL pellet using Shandon Cytospin (Thermo Shandon, Inc., Pittsburg, PA). Following centrifugation, the slides were stained with the Diff‐Quik kit (Dade Behring Inc., Newark, Delaware). Cell differential was performed by counting macrophages/monocytes, neutrophils, eosinophils from 500 cells on each slide using brightfield microscopy.

### 2.5 VEA chemical analysis of BALF

All chemical analyses were performed blind. An equal volume of hexane to BALF supernatant was used for liquid–liquid extraction. The organic phase was decanted and used for GC‐MS analysis. VEA from the BALF samples were analyzed using an Agilent 6890 N gas chromatograph coupled to an Agilent 5973 N quadrupole mass spectrometer (GC‐MS, Agilent Technologies Inc., Santa Clara, CA). Electron ionization mass spectra for VEA were greater than 90% matched to the database of the National Institute of Standards and Technology (U.S. Food and Drug Administration, 2020). Calibrations for VEA had a *R* ^2^ of 0.99. Calibrations were performed in triplicate for human variation (about 4%) and injected in triplicate for instrument variation (about 7% for VEA). Extraction efficiency was performed by spiking known amounts of VEA into BALF fluid. Extraction efficiency was >95%. The error for the extraction was approximately 3%–4%.

### 2.6 Lung tissue collection

The left lungs were inflation‐fixed with 4% paraformaldehyde under 30 cm of hydrostatic pressure for 1 h. After inflation, the left lungs were stored in 4% paraformaldehyde for 24 h and transferred into 70% ethanol for additional fixation at 4°C. Each left lung was cut into four transverse slices, processed in the Authotechnicon (Shandon Citadel 1000, Thermo Fisher, Pittsburgh, PA), then embedded in paraffin (Surgipath Plus, Leica Biosystems, Deer Park, IL). The left lung blocks were cut into 5 μm sections using a rotary microtome (HM 355, Microm, Walldorf, Germany) and mounted onto Permafrost plus slides (Fisher Scientific, Pittsburgh, PA). The slides were deparaffinized and stained with hematoxylin and eosin (American MasterTech, Inc., Lodi, CA) for histopathology.

### 2.7 Semiquantitative lung histopathology scoring

Histopathology of the left lungs was evaluated using a semi‐quantitative scoring system as previously described (Castañeda & Pinkerton, 2016; D'Evelyn et al., 2023; Yuan et al., 2022). In brief, each region of the lung (airways, blood vessels, alveoli, and pleura) was scored based on the product of the severity (0–3) and the extent (0–3) of inflammation present. The overall histopathology (injury) of the lungs was calculated using the sum of the scores from each region of the lungs (airways, blood vessels, alveoli, and pleura).

### 2.8 Immunohistochemistry and quantification

Paraffin sections of the left lungs were deparaffinized, rehydrated and heated in diluted 10X EDTA (Sigma Aldrich, Saint Louis, MO) using a decloaker (Biocare Medical; Pacheco CA) to retrieve antigens. Following antigen retrieval, slides were treated with 3% hydrogen peroxide and protein block (Cat. NO. X0909, DAKO; Agilent, Santa Clara, CA). Club cell secretory protein was labeled by 1‐h incubation of 1:10,000 rabbit anti‐mouse club cell secretory protein (CCSP) antibodies (Cat. NO. ab213203, Abcam, Waltham, MA). The labeled CCSP was stained by 1‐h incubation of EnVision+ System anti‐rabbit HRP labeled polymer (Cat. NO. K4003, DAKO; Agilent, Santa Clara, CA) and 5‐min incubation of DAB (Cat. NO. K5007, DAKO; Agilent, Santa Clara, CA).

The CCSP was quantified in the intrapulmonary lobar bronchus of the left lung. The total surface area of the airway epithelium and the surface area containing CCSP positive staining of the airway epithelium were quantified using the ImageJ program (public domain). The proportion of CCSP‐positive epithelium was calculated from the area of CCSP‐positive epithelium divided by the total surface area. For each animal, the proportion was calculated from the mean of four different fields.

### 2.9 RT‐qPCR

RNA from the middle and accessory lobes of the right lungs were extracted using Trizol (Cat. NO. 15596018, Invitrogen, Waltham, MA) chloroform extraction. The initial extraction was further purified using a miniprep RNA extraction kit (Cat. NO. R1055, Zymo Research, Irvine, CA). The purified RNA was amplified to 1 μg cDNA using a high‐capacity cDNA Reverse Transcription Kit (Cat. NO. R1055, Applied Biosystem, Waltham, MA). Detection of β‐actin as a housekeeping gene and differentially expressed target genes was performed with a LightCycler LC480 Instrument (Roche Diagnostics, Indianapolis, IN, USA) using the Fast SYBR Green Master Mix (Cat. NO. 4309155, Applied Biosystems, Waltham, MA) according to the manufacturer's instructions. Gene expression was quantified using the ΔΔ‐Ct method and normalized by β*‐actin* (*Actb*).

### 2.10 Serum sRAGE quantification

The whole blood was collected from the right ventricles of the animals during the necropsy. Serum sRAGE levels were quantified using the murine sRAGE Quantikine ELISA kit (Cat. NO. MRG00, R&D Systems, Minneapolis, MN, USA) following the manufacturer's instructions.

### 2.11 Statistical analysis

Analysis of variance (ANOVA) was performed using Prism version 10.5.0 (Graphpad, Boston, MA). Log transformations were performed on datasets with a right‐skewed normal distribution. A 2‐way ANOVA (female vs. male, VEA vs. filtered air) was used to analyze the vital measures and CCSP coverage in the 10‐day exposures. A 3‐way ANOVA model was used to test for statistical differences between sex (female vs. male), duration of exposure (3 days vs. 10 days), and treatment (VEA aerosol vs. filtered air). Tukey's multiple comparison was performed using a *p* value of 0.05. All values were described or displayed as mean ± standard error.

## 3 RESULTS

### 3.1 VEA aerosol concentrations in the exposure chamber

In the 3‐day exposures, the daily average chamber aerosol concentration of the male and female mice was 890 ± 123 mg/m^3^ and 1194 ± 91 mg/m^3^, respectively. The 10‐day average aerosol chamber concentration of the male and female mice was 749 ± 81 mg/m^3^ and 693 ± 52 mg/m^3^, respectively. Within each exposure duration, there was no statistical difference in the average chamber concentration between males and females. When compared regardless of exposure duration, only the chamber aerosol concentration of the 3‐day females was significantly higher compared to its 10‐day counterpart.

### 3.2 Vital measurements

**FIGURE 1:**
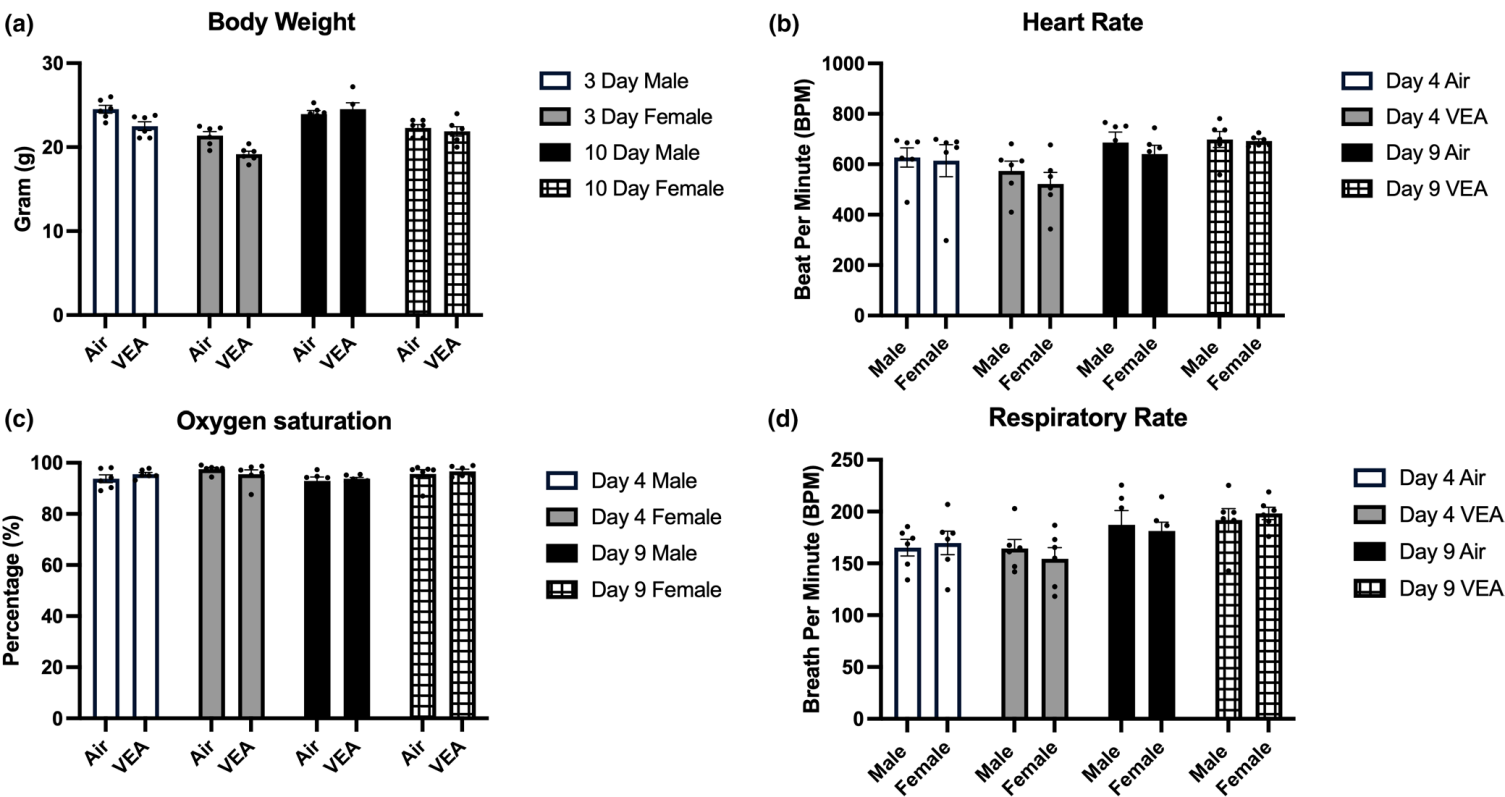
Physiological parameters of animals are presented as mean ± standard error (SE), stratified by exposure condition (VEA or Air), exposure length, and sex. (a) Body weight measured on the day prior to necropsy for animals in 3‐day and 10‐day exposure groups revealed no statistical difference when compared to control groups. Similarly, (b) heart rate (BPM), (c) oxygen saturation (%), and (d) respiratory rate (BRPM) measured for animals on Days 4 and 9 of the 10‐day exposure group. No statistically significant differences were noted between VEA‐exposed animals and filtered air controls. *n* = 6/treatment group. BPM, beats per minute; BRPM, breaths per minute; VEA, vitamin E acetate.

The average body weight of the VEA‐exposed mice shows no statistical difference compared to the control groups in the 3‐day and 10‐day exposures (Figure 1a). In addition, there was no statistical difference in average heart rate (Figure 1b), oxygen saturation (Figure 1c), or respiratory rate (Figure 1d) between the VEA‐exposed and the control groups on Day 4 or Day 9 during the 10‐day exposure. When males and females are compared, no pair of groups demonstrates statistical differences in any of these measurements.

### 3.3 BALF chemical analysis

VEA was detected in the BALF of all four VEA treatment groups. Following 3 days of exposure, VEA in the BALF of males and females was 9.2 ± 2.4 μg/mL and 7.6 ± 1.2 μg/mL, respectively. Following 10 days of exposure, VEA concentrations in males and females were 20.4 ± 4.0 μg/mL and 5.5 ± 1.6 μg/mL, respectively. BALF from the 10‐day VEA male mice had the highest concentration of VEA, which was significantly higher compared to the females with the same duration of exposure. VEA was not detected in the BALF of 3‐ and 10‐day control mice.

### 3.4 BAL cell counts and differentials

When males and females are combined in each exposure duration, the 10‐day VEA group displayed significant increases in total cells, macrophages, neutrophils, and lymphocytes compared to its respective control. There is no statistical difference between the 3‐day VEA and its respective control.

**FIGURE 2:**
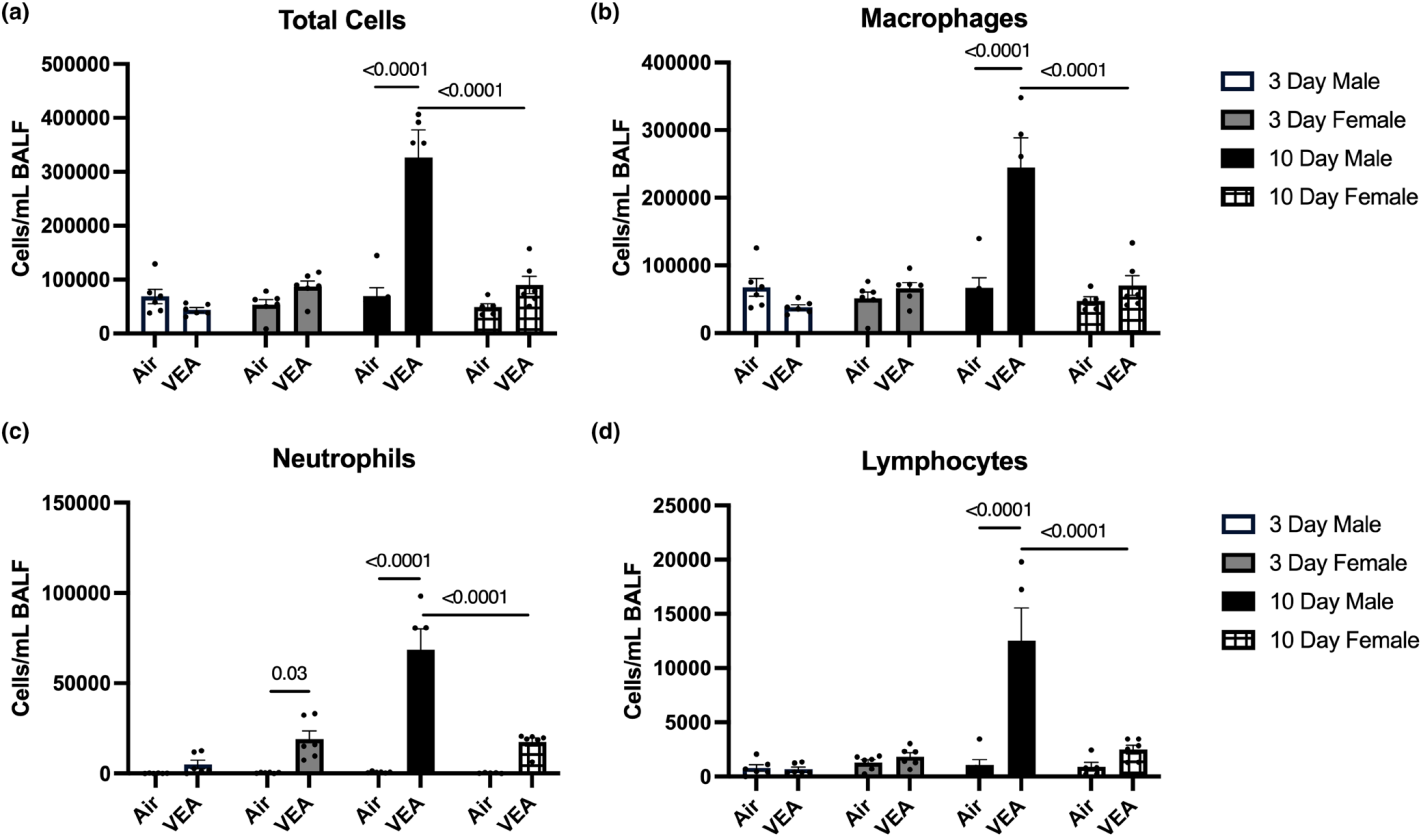
Following 3‐day and 10‐day exposures to VEA or to filtered air, bronchoalveolar lavage fluid (BALF) was collected and pelleted for analysis of (a) total cells, (b) macrophages, (c) neutrophils, and (d) lymphocytes. 10‐day VEA‐exposed males exhibited a significant increase in total cells, macrophages, and neutrophils when compared to the respective controls. Despite no statistical significance following the 3‐day VEA exposure in males or female mice when compared to control groups, 10‐day VEA‐exposed males were found to have significantly greater quantities of total cells, macrophages, neutrophils, and lymphocytes when compared to the 10‐day and 3‐day VEA‐exposed females. Column height represents mean cells/mL BALF ± standard error (SE). *N* = 6/treatment group. BALF, bronchoalveolar lavage fluid; VEA, vitamin E acetate.

Males and females showed different increments in differentials. Compared to the female VEA groups, the 10‐day VEA male had significantly higher numbers of total cells (Figure 2a), macrophages (Figure 2b), neutrophils (Figure 2c), and lymphocytes (Figure 2d) compared to its respective control while these increases were not observed in the 10‐day female.

### 3.5 BALF protein levels

Overall, both the 3‐day and 10‐day VEA groups showed significantly higher total protein levels compared to their respective sham controls. In addition, 3‐day VEA had significantly higher protein levels compared to the 10‐day counterpart.

**FIGURE 3:**
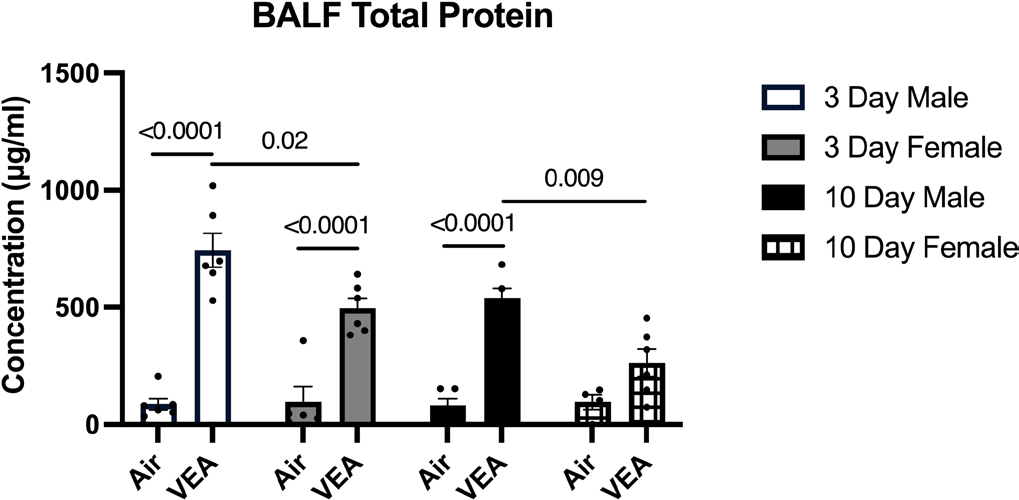
Total protein concentration (μg/mL) in the BALF supernatant is indicated as mean ± standard error (SE), stratified by exposure condition (VEA or Air), exposure length, and sex. Overall, all VEA‐exposed groups displayed significantly higher levels of protein in comparison to their respective controls. Yet, the 3‐day VEA‐exposed males presented with the greatest protein levels when compared to all VEA‐exposed females. *N* = 6/treatment group. BALF, bronchoalveolar lavage fluid; VEA, vitamin E acetate.

When stratified by sex, all four VEA groups showed significantly higher total protein levels compared to their respective control (Figure 3). In addition, the 3‐day VEA male showed the highest protein levels compared to either the 3‐ or 10‐day VEA female group. The average protein level of the male VEA 10‐day was also significantly higher compared to the female counterpart.

### 3.6 Semiquantitative histopathologic scoring

**FIGURE 4:**
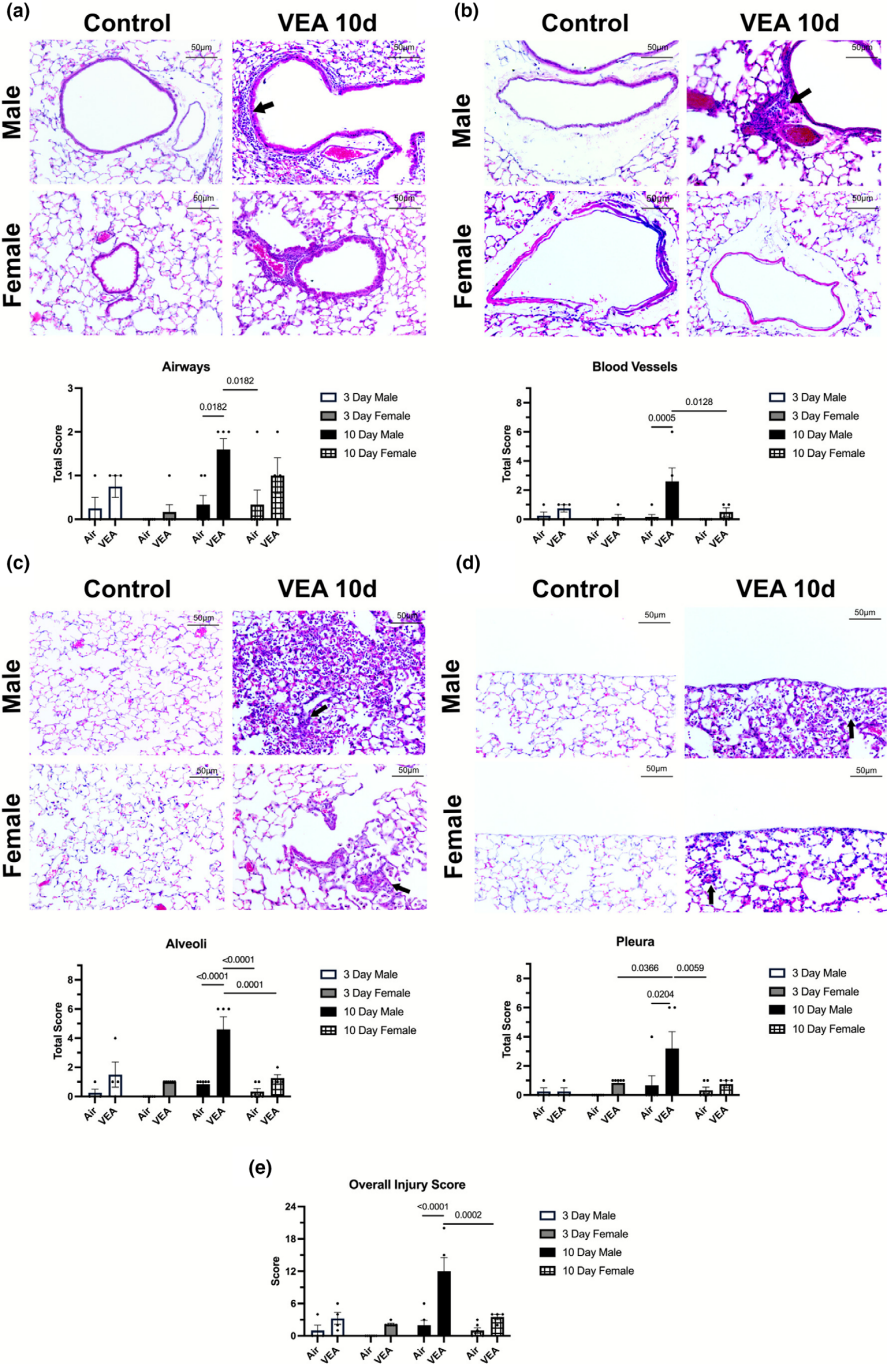
Hematoxylin and eosin staining of lung tissue from 10‐day control and VEA‐exposed mice reveals alterations to (a) airway peribronchiolar, (b) blood vessel perivascular, (c) alveolar, and (d) pleural spaces of the lung. Representative light microscopy images (control, left column; VEA 10 days, right column; male, top row; female, bottom row) display (a) a significant increase in cell aggregates (arrow) surrounding airways (peribronchiolar) in males; (b) heightened immune cell recruitment (arrow) around blood vessels (perivascular) in males compared to females; (c) alveoli air spaces with a significantly increased influx of focal agglomerates of leukocytes (arrow) in males compared to females; and (d) increased inflammation (arrow) in the pleural region in both males and females. All images were taken using a 20× objective lens. Semiquantitative histology scores of the lungs in male and female mice were completed by two independent observers following 3‐day and 10‐day exposure to VEA or to filtered air. This comprehensive analysis comparing 10‐day VEA‐exposed males and females to their respective controls revealed significantly increased (b) bronchiolar, (b) perivascular, (c) alveolar, (d) pleural inflammatory scores, as well as (e) a total lung injury score in males compared to filtered air control males. In contrast, no statistically significant increases in inflammation were present in VEA‐exposed females, compared to filtered air controls. These semiquantitative scores (severity × extent) could range from 0 to 9, while the overall lung injury (the combined scores from each of the four regions) could range from 0 to 36. Data is presented as mean score ± standard error (SE). *N* = 6/treatment group. VEA, vitamin E acetate.

The combined data of both males and females showed significantly higher histopathology scores in airways (peribronchiolar), blood vessels (perivascular), alveoli, pleural, and overall lung injury following 10 days of VEA exposure, but not following 3 days of exposure. The 10‐day VEA male group showed significantly higher scores in airways (Figure 4a), blood vessels (Figure 4b), alveolar (Figure 4c), pleural (Figure 4d), and the total (Figure 4d) relative to its control. In contrast, the 10‐day VEA female did not have significantly higher inflammation scores in any region compared to its respective controls.

### 3.7 CCSP immunohistochemistry

**FIGURE 5:**
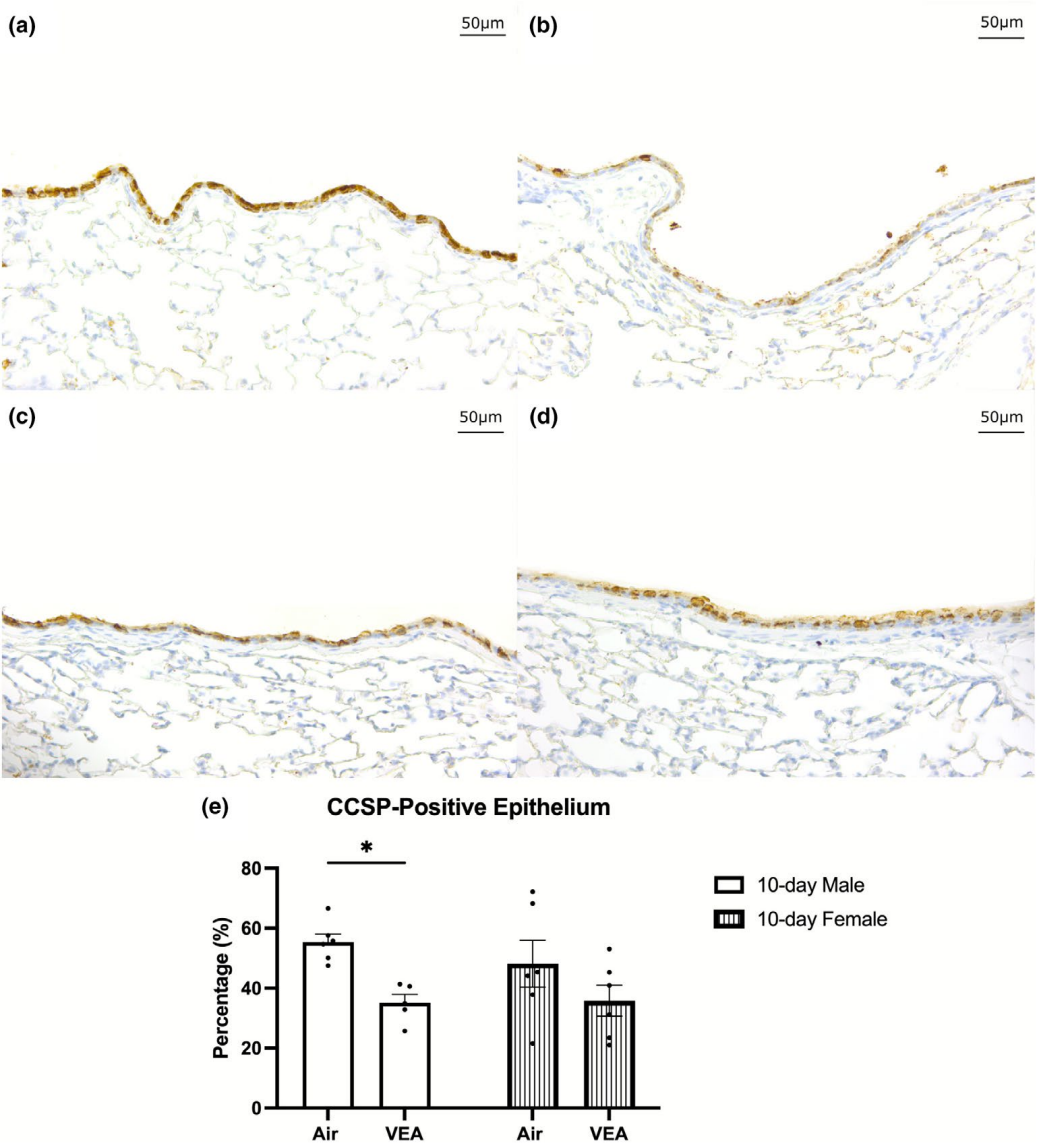
CCSP immunostaining of the left intrapulmonary lobar airway was performed in mice exposed for 10 days to VEA or to filtered air. VEA exposure was associated with a significant reduction of CCSP‐positive airway epithelium compared to the control group in males. Females also demonstrated a reduction in CCSP immunostaining of the airway epithelium, although no statistically significant difference was noted between 10‐day VEA‐exposed females to the filtered air control. Representative light microscopy images (control, left column; VEA 10 days, right column; male, top row; female, bottom row) reveal significant CCSP reduction in male airway epithelium. All images were taken using a 20× objective lens. The bar graphs represent the mean percent of the total airway epithelium staining positive for CCSP as the average ± standard error (SEN = 6/treatment group. CCSP, club cell secretory protein; VEA, vitamin E acetate.

When males and females are combined, there is a significant reduction of CCSP‐positive airway epithelium in the 10‐day VEA group compared to its control. In contrast, when stratified by sex, results showed only a significant reduction in CCSP expression among males but not females (Figure 5).

### 3.8 Gene expression

When males and females are combined in each exposure duration, the 10‐day VEA group showed significantly higher expression in interleukin 1 beta (*Il1b*), interleukin 6 (*Il‐6*), keratinocyte chemoattractant (*Kc*), and C‐X‐C motif chemoattractant 5 (*Cxcl5*) compared to its respective control. In addition, the 10‐day combined VEA group showed significantly higher expression in all four genes compared to the 3‐day combined counterpart.

**FIGURE 6:**
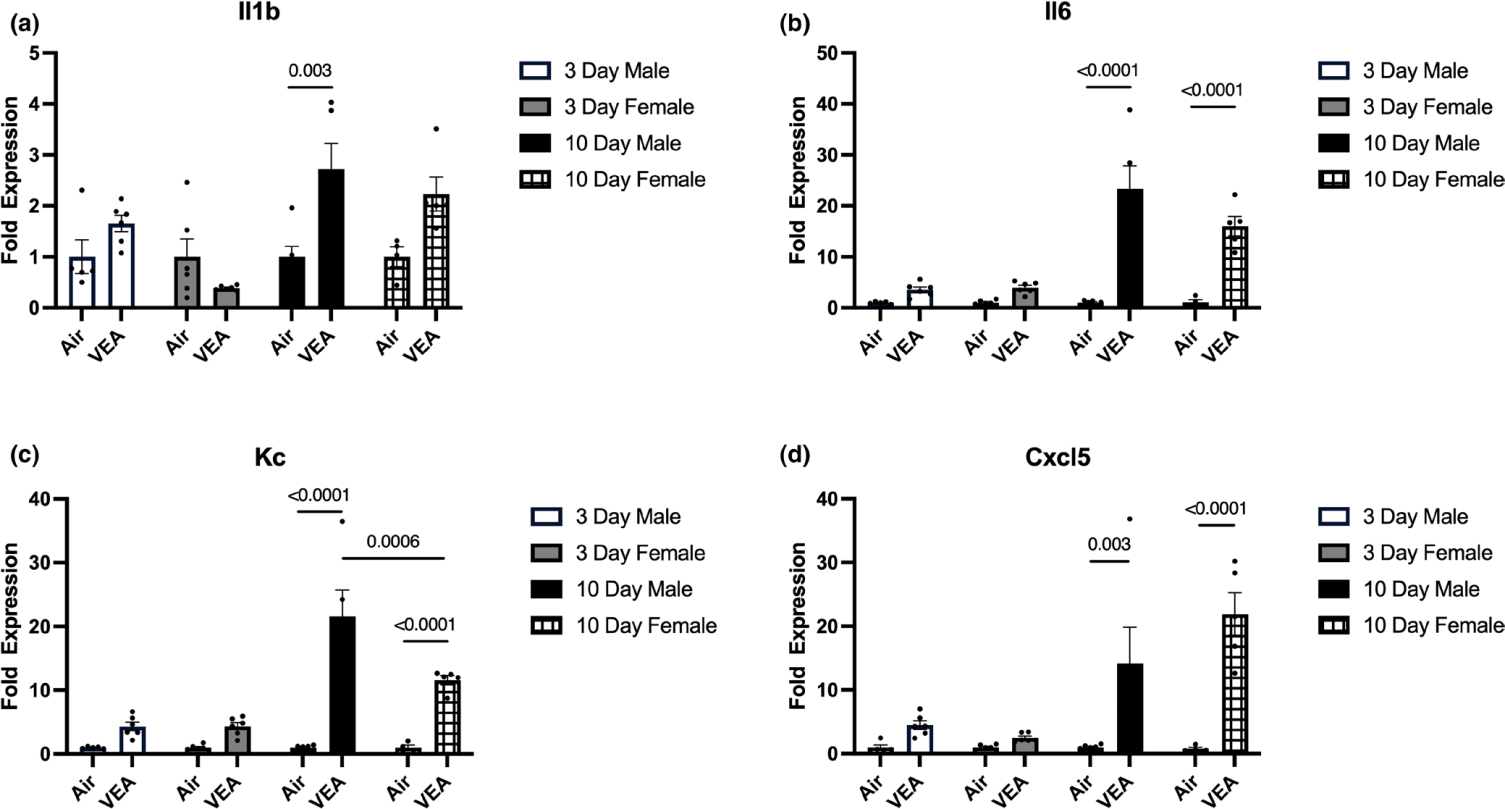
Exposure to VEA modifies mRNA fold expression levels. 10‐day VEA‐exposed males and females presented with significantly increased expression of (a) IL‐1β, (b) IL‐6, (c) Kc, and (d) Cxcl5 from right lung homogenates compared to the respective control groups, despite no statistical significance of 3‐day VEA‐exposed males and females. Although a statistically significant increase in (a) IL‐1β expression was observed in the 10‐day VEA‐exposed males, the increase was not significant in the female mice. Expression of (c) Kc in 10‐day exposed males was significantly greater compared to their female counterparts. While not significant, (b) IL‐6 expression was greater, and (d) Cxcl5 exhibited lower expression in males compared to females. Bars represent mean ± standard error (SE). *N* = 6/treatment group. Cxcl5, C‐X‐C motif chemoattractant 5; IL‐1β, interleukin‐1 beta; IL‐6, interleukin‐6; Kc, keratinocyte chemoattractant; VEA, vitamin E acetate.

When stratified by sex, VEA males and females showed no statistical difference in gene expressions in the 3‐day exposure. In the 10‐day exposure, however, males and females exhibit differential expressions of proinflammatory cytokines. The 10‐day VEA male showed a significant increase in *Il1‐b* expression compared to its control while such an increase was not significant in the female counterpart (Figure 6a). *Il‐6* expression was higher in the male compared to the female, but this difference was not statistically significant (Figure 6b). *Kc* expression in the male was significantly higher compared to the female counterpart (Figure 6c). *Cxcl5* expression was lower in the male compared to the female, but this difference was not statistically significant (Figure 6d).

### 3.9 Serum sRAGE quantification

Combined data showed significantly higher serum sRAGE levels in both the 3‐day and 10‐day VEA groups compared to their respective controls. In addition, the sRAGE level in the 10‐day VEA was significantly higher than that of the 3‐day VEA.

**FIGURE 7:**
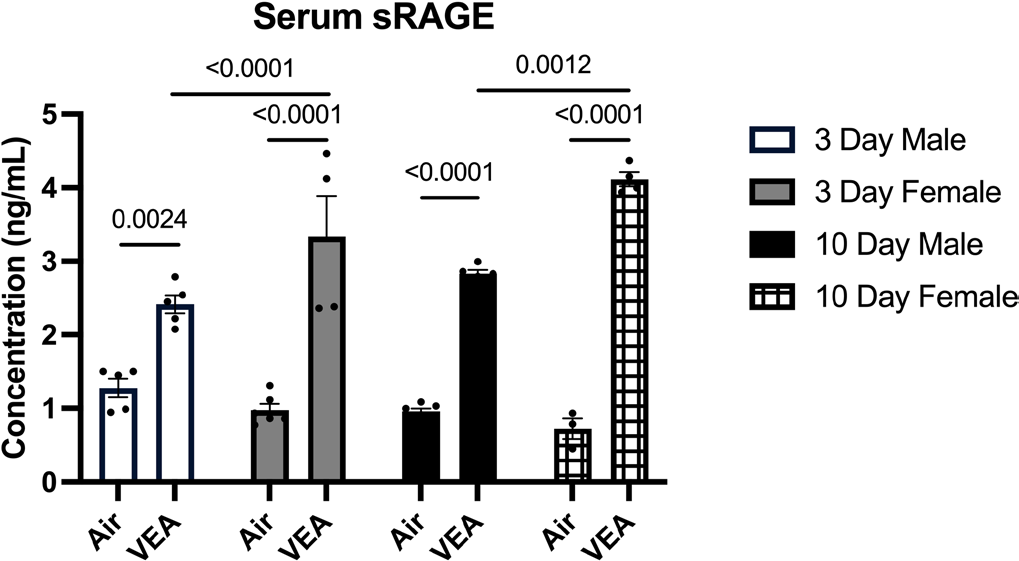
Serum sRAGE concentration (ng/mL) in the BALF supernatant was measured using ELISA. 3‐day and 10‐day VEA‐exposed males and females displayed significantly higher serum sRAGE levels compared to control groups. Additionally, 3‐day and 10‐day VEA‐exposed females presented with significantly higher sRAGE levels when compared to their male counterparts. Column height represents mean concentration ± standard error (SE). *N* = 6/treatment group. BALF, bronchoalveolar lavage fluid; sRAGE, soluble receptor of advanced glycation end products; VEA, vitamin E acetate.

When stratified by sex, both the 3‐day VEA male and female had significantly higher sRAGE concentrations compared to their respective controls (Figure 7). The level of the 3‐day VEA female was significantly higher than the male counterpart. In the 10‐day exposure, male and female also had significantly higher sRAGE concentrations compared to their respective controls. Further, the VEA female had a higher sRAGE concentration compared to the VEA male.

## 4 DISCUSSION

We developed a novel young‐adult murine model of subacute VEA exposure to investigate the temporal relationship between recreational use and lung injury onset and to examine sex‐stratified pathobiological responses. After 10 days, VEA‐exposed mice exhibited acute features of pathologic lung injury, inflammation, and higher systemic plasma sRAGE levels. CCSP expression was reduced in large airways, most prominently in males. Notably, early changes in alveolar barrier integrity were evident as soon as 3 days post‐exposure, with increased BAL neutrophils, total protein, and plasma sRAGE despite the absence of overt histopathology. Sex‐stratified analyses showed greater histologic lung injury and immune dysregulation in males, with elevated Il1b, Il6, and Kc expression, while females had higher sRAGE levels despite milder pathology. Notably, all mice maintained stable vital signs consistent with normative murine values under basal conditions (Dutta & Sengupta, 2016), even with the pronounced histologic changes observed after 10 days of repeated VEA exposure. This finding suggests an indolent disease trajectory, with clinical manifestations likely emerging only at later stages.

Despite comparable aerochamber VEA concentrations and anatomic size, male mice exhibited markedly higher BALF VEA levels over time, with significant elevations relative to females by day 10. These findings align with prior in vivo studies demonstrating the pulmonary toxicity of aerosolized VEA in short‐term (6–28 days), intermittent (1–5 h/day) exposure models designed to mimic recreational vaping (Castañeda & Pinkerton, 2016; Dutta & Sengupta, 2016; Lim et al., 2021; Raucci et al., 2008). Histopathological analyses in these models have revealed oil‐laden macrophages, indicative of VEA accumulation and macrophage activation, and interstitial inflammation consistent with inflammatory lung injury. Complementary in vitro studies show that human alveolar type II (ATII) cells sustain direct VEA‐induced injury, accompanied by increased release of monocyte‐ and neutrophil‐recruiting chemokines. The elevated BALF VEA concentrations observed here likely reflect the severity of pulmonary injury and associated impaired alveolar fluid clearance, while alterations in pulmonary metabolism may also further contribute to heightened, sex‐specific susceptibility, warranting additional investigation.

One of the most compelling findings from our study is the distinct plasma sRAGE profiles observed between male and female mice. RAGE is a transmembrane, multiligand receptor constitutively expressed on type I alveolar epithelial cells and inducibly on alveolar endothelial cells (Lim et al., 2021). Under oxidative stress, RAGE activation triggers intracellular signal transduction via NF‐κB, driving a cascade of pro‐inflammatory cytokine release and tissue injury. Its soluble isoform (sRAGE), generated through alternative splicing or proteolytic cleavage, acts as both a biomarker of RAGE activation and a decoy receptor that dampens ligand‐induced inflammatory signaling (Braley et al., 2016; Lim et al., 2021; Raucci et al., 2008; Sukkar et al., 2012). Although sex‐dependent hormonal regulation of the RAGE pathway has been described in a variety of non‐pulmonary diseases (Bajwa et al., 2023; Fernandez et al., 2010; Mukherjee et al., 2005; Tanaka et al., 2000; Tesarová et al., 2007), we are the first to report sex‐dimorphic sRAGE responses in an EVALI model and, to our knowledge, in any acute lung injury model. It is plausible that estrogen may enhance ADAM10‐mediated RAGE shedding, driving higher circulating sRAGE in females and modulating sex‐specific lung injury (Fernandez et al., 2010). Sex‐specific differences in CCSP expression along the large airway epithelium were also observed, with CCSP also serving a critical immunomodulatory role and essential to epithelial repair (Almuntashiri et al., 2023; Martinu et al., 2023). Together, these findings highlight potential hormonally regulated pathways that may define sex‐specific vulnerability and inform targeted therapies for VEA‐induced lung injury.

Intrinsic sex differences modulate the pathophysiology, incidence, morbidity, and mortality of multiple lung diseases across the lifespan, ranging from neonatal conditions to adult diseases that include asthma, cystic fibrosis, idiopathic pulmonary fibrosis, chronic obstructive pulmonary disease, and lung cancer (Silveyra et al., 2021). Male‐biased outcomes have also been observed in viral epidemics, including COVID‐19, SARS‐CoV‐2, and MERS‐CoV (Matute‐Bello et al., 2008). The underlying causes of these differences remain unclear, and it is not fully established to what extent they arise from biological versus environmental factors. In this model, we demonstrate that biological sex differences in EVALI are partly mediated by differential immune dysregulation, likely influenced by hormonally regulated pathways, which may contribute to the sex‐specific disparities observed across the spectrum of lung diseases.

Our study offers several strengths and contributes to the limited body of literature on VEA‐induced lung injury. A major innovation of our model is the balanced inclusion of male and female mice, enabling direct investigation of sex‐based differences in pulmonary response. To our knowledge, this is the first animal model to specifically interrogate biological sex as a determinant of the dysregulated immune response to inhalational VEA exposure. Second, biomarkers of disease remain underexplored in the context of EVALI. Building upon a limited body of mechanistic VEA literature, our study identifies biomarkers of neutrophil‐dependent lung injury, pro‐inflammatory signaling, and epithelial damage, providing new insights into core mechanisms of injury and highlighting emerging therapeutic targets for future investigation. We focused specifically on a young adult mouse model, representing an important demographic with EVALI. This subgroup is known to exhibit a distinct pathophysiologic response compared to older adults, making it particularly relevant for modeling age‐specific disease dynamics. Finally, our study investigated early changes in biomarker profiles and identified evidence of evolving lung injury as early as 3 days following VEA inhalation exposure, equivalent to roughly 3 months of human exposure (Dutta & Sengupta, 2016). These findings underscore the rapid onset of pulmonary toxic effects of recreational VEA use among adolescents and young adults, extending beyond prior literature and signaling a significant public health risk.

Limitations to our study include the fact that while experimental murine models are widely used to investigate human disease, mice differ in anatomy and the innate immune response. Notably, murine toll‐like receptor 4 signaling diverges from humans, and mice lack the CXCL8 (IL‐8) gene, instead producing functional analogs KC (CXCL1) and MIP‐2 (CXCL2) (Matute‐Bello et al., 2008, 2011). Although we used an inbred strain (C57BL/6) mice to limit genetic variability (homozygous at almost all loci), the sex‐stratified analysis did not employ exclusively sex‐matched littermates, which may introduce potential environmental and genetic variability. In addition, some variability was observed in the chamber concentrations of VEA, specifically between the 3‐day and 10‐day female groups, although male versus female exposure levels were not significantly different within each timepoint. Inconsistent aerosol generation by heating coils may explain this variation, especially in the 3‐day female group. While the exposure regimen (3 h/day on weekdays using a third‐generation e‐cigarette) was designed to reflect real‐world standards of operating use, human‐equivalent exposure may still differ. Nonetheless, stable murine vital signs and consistent body weight suggest reliable minute ventilation and delivery, as well as exposure within typical thresholds of exposure. Another limitation is the use of pure VEA, which does not fully replicate the complex chemical matrix of THC/VEA‐containing aerosols; real‐world mixtures may produce synergistic effects on both bioactivity and toxicity not captured here. Furthermore, we did not examine a dose–response range of VEA exposure or the precise mechanisms of VEA‐induced lung injury. While prior in vivo studies have implicated direct cytotoxic effects of VEA on alveolar epithelial cells and hydrolysis of VEA to free tocopherol triggering oxidative stress along the airways (Matsumoto et al., 2022), the full spectrum of injury remains unclear. These may include interference of hydrophobic VEA particles with pulmonary surfactant, the presence of heavy metals in the inhaled aerosol, or thermal degradation of VEA into reactive compounds such as ketene at vaporization temperatures (Massey et al., 1982; Rebuli et al., 2023; Wu & O'Shea, 2020). Lastly, we did not explore sex‐specific molecular pathways beyond the biomarkers assessed, with the role of estrogen mediation on the RAGE axis warranting further investigation.

Following the outbreak of EVALI cases in 2019 and mounting evidence implicating VEA as a primary toxic agent, the FDA advised against the use of VEA in any vaping or e‐cigarette products (U.S. Food and Drug Administration, 2020). Despite this, clinical reports continue to detect VEA in patients with EVALI (Callahan et al., 2024). Although cannabis legalization has increased access to regulated THC products, it may also be inadvertently fueling the illicit market for contaminated e‐liquids and counterfeit cartridges. In a recent review of 15,748 published cases across 161 medical reports of EVALI, 65% of patients were male and 35% were female. Age distribution showed 80% of the EVALI population is under the age of 34 years, with 17% aged between 13 and 17 years of age (Marrocco et al., 2022). Clinical manifestations are nonspecific (Cao et al., 2020), with no validated diagnostic biomarkers or precision‐based therapies available, and the long‐term health consequences of use remain unknown, underscoring a pressing need for mechanistic and translational research (Rebuli et al., 2023).

In conclusion, VEA remains a significant causative agent of EVALI and continues to be a major public health concern. In this adolescent animal model of subacute VEA exposure, we observed histopathologic lung tissue injury and dysregulated inflammation at 10 days of exposure, alongside signs of alveolar barrier dysfunction as early as 3 days, despite the absence of overt vital derangements. Male mice comprised a distinct subgroup exhibiting heightened susceptibility to worsened injury, with sex‐specific differences in immune response, CCSP, and sRAGE expression. To our knowledge, this study is the first to identify sexual dimorphism in lung disease manifestation following short‐term inhaled VEA exposure. These findings may enhance diagnostic precision, inform targeted treatment development, and further advance mechanistic understanding of EVALI pathogenesis. Recognizing sex as a relevant biological variable is essential to understanding the heterogeneity of EVALI‐associated outcomes. A sex‐informed approach to future research may further uncover critical features of immune‐pathogenesis associated with EVALI and further refine public health strategies aimed at mitigating use and exposure, particularly among high‐risk populations.

## AUTHOR CONTRIBUTIONS

ML and XL are co‐first authors and contributed to the research work equally. Conceived and designed research: ML, XL, JY, ES, and KP. Analyzed data: XL and JY. Performed experiments: ML, XL, JY, HK, DS, NE, LT, TN, and KP. Interpreted results of experiments: ML, XL, HK, JL, and KP. Prepared figures: XL, JY, HK, and JL. Drafted manuscript: ML, XL, and JY. Edited and revised the manuscript: ML, XL, KP, JL, TA, MA, KP, and MM. Approved final version of the manuscript: ML, XL, JY, HK, DS, NE, LT, TN, JL, ES, TA, MA, KP, and MM.

## CONFLICT OF INTEREST STATEMENT

None.

## ETHICS STATEMENT

All animal procedures were approved by the UC Davis Institutional Animal Care and Use Committee (IACUC).

## Data Availability

The data that support the findings of this study are available from the corresponding author upon reasonable request.
